# National Landscape Assessment of Sexual and Gender Minority Inclusivity of the VA’s Home Based Primary Care Services

**DOI:** 10.1093/geroni/igaf122.1815

**Published:** 2025-12-31

**Authors:** Joy Lee, Michelle Hilgeman, Tetla Menen, Nicholas Sweeney, Lorraine Stigar, Jennifer Carnahan

**Affiliations:** Mass General Brigham, Boston, Massachusetts, United States; Tuscaloosa VA Medical Center, Tuscaloosa, Alabama, United States; Roudebush VA Medical Center, Indianapolis, Indiana, United States; University of Massachusetts Chan Medical School, Worcester, Massachusetts, United States; Purdue University, West Lafayette, Indiana, United States; Indiana University Center for Aging Research, Indianapolis, Indiana, United States

## Abstract

**Background:**

Sexual and gender minority (SGM) Veterans are more likely to require home care services than non-SGM Veterans. However, fear of discrimination deters many from seeking these supports. This landscape analysis seeks to assess team readiness and experiences with older SGM veterans within the U.S. Department of Veterans Affairs’ (VA) Home Based Primary Care (HBPC) program.

**Methods:**

Using a multi-method approach, we conducted a survey of VA HBPC staff nationwide and five focus groups with staff at select sites.

**Results:**

Of approximately 10,000 HBPC staff invited to participate, 321 responded. Respondents included clinicians (44%; e.g., nurses and physicians), social workers (20%), and other staff (31%; e.g., nutritionists). Most reported minimal training on SGM care, with over half (54%) receiving less than three hours in the past five years; 12% received no training at all. Despite this, 43% strongly agreed they were aware of the unique challenges faced by SGM Veterans and 46% somewhat agreed. Regarding community resources for SGM Veterans, 71% endorsed confidence in finding facilities safe from discrimination, but only 61% endorsed confidence finding an affirming one. In focus groups, staff reported limited experience with SGM veterans and raised concerns about the inclusivity of community resources. They also noted a strong desire for training in SGM care.

**Conclusion:**

Our landscape assessment found that HBPC staff had concerns about the inclusivity and affirming nature of both VA and external services. Further training and resources are needed to enable the VA to deliver exemplary, affirming care for older SGM Veterans.

